# Enhanced Human Neutrophil Vitamin C Status, Chemotaxis and Oxidant Generation Following Dietary Supplementation with Vitamin C-Rich *SunGold* Kiwifruit

**DOI:** 10.3390/nu7042574

**Published:** 2015-04-09

**Authors:** Stephanie M. Bozonet, Anitra C. Carr, Juliet M. Pullar, Margreet C. M. Vissers

**Affiliations:** Centre for Free Radical Research, Department of Pathology, University of Otago, Christchurch, PO Box 4345, Christchurch 8140, New Zealand; E-Mails: anitra.carr@otago.ac.nz (A.C.C.); juliet.pullar@otago.ac.nz (J.M.P.); margreet.vissers@otago.ac.nz (M.C.M.V.)

**Keywords:** neutrophil, vitamin C, ascorbate, chemotaxis, oxidants, NETs, apoptosis

## Abstract

Neutrophils are the body’s primary defenders against invading pathogens. These cells migrate to loci of infection where they engulf micro-organisms and subject them to an array of reactive oxygen species and antimicrobial proteins to effect killing. Spent neutrophils subsequently undergo apoptosis and are cleared by macrophages, thereby resolving the inflammatory episode. Neutrophils contain high concentrations of vitamin C (ascorbate) and this is thought to be essential for their function. This may be one mechanism whereby vitamin C enhances immune function. The aim of our study was to assess the effect of dietary supplementation with vitamin C-rich *SunGold* kiwifruit on four important functions of neutrophils: chemotaxis, oxidant generation, extracellular trap formation, and apoptosis. Fourteen young men (aged 18–30 years) with suboptimal plasma vitamin C status (<50 μmol/L) were supplemented for four weeks with two *SunGold* kiwifruit/day. Plasma vitamin C status was monitored weekly and neutrophil vitamin C levels were assessed at baseline and post-intervention. Neutrophil function assays were carried out on cells isolated at baseline and post-intervention. Plasma vitamin C levels increased to >70 μmol/L (*p* < 0.001) within one week of supplementation and there was a significant increase in neutrophil vitamin C status following four weeks’ intervention (*p* = 0.016). We observed a significant 20% increase in neutrophil chemotaxis post-intervention (*p* = 0.041) and also a comparable increase in oxidant generation (*p* = 0.031). Supplementation did not affect neutrophil extracellular trap formation or spontaneous apoptosis. Our data indicate that supplementation with vitamin C-rich kiwifruit is associated with improvement of important neutrophil functions, which would be expected to translate into enhanced immunity.

## 1. Introduction

Vitamin C (ascorbate) is made by plants and most animals, but humans have lost this ability due to mutation of the gene encoding l-gulono-γ-lactone oxidase, the final enzyme in the biosynthetic pathway [1]. Thus, vitamin C is an essential micronutrient for humans and must be obtained on a daily basis, through the diet, in order to prevent hypovitaminosis C and the life-threatening deficiency disease scurvy. Plasma levels of vitamin C reflect dietary intake and typically vary between 20 and 80 μmol/L [2], however, active uptake by circulating leukocytes and tissues results in intracellular concentrations that are 10–100 fold higher than plasma [3]. Neutrophils accumulate vitamin C via the sodium-dependent vitamin C transporter 2 (SVCT2) [4] and have intracellular levels of 1–2 mM [5]. However, during their oxidative burst, when neutrophils produce highly reactive oxidants to combat invading pathogens, they increase their intracellular concentration of vitamin C through the non-specific uptake of the oxidised form, (dehydroascorbate, DHA) via the glucose transporters [5,6]. DHA is then reduced to ascorbate intracellularly, to give levels of up to 10–20 mM following stimulation. It is generally believed that the accumulation of such high vitamin C concentrations indicates an essential function within these cells, although these functions remain unidentified.

Neutrophils are one of the first cellular responders to inflammatory challenge. Their primary function is to destroy invading microorganisms and thereby prevent systemic infection. They phagocytose microorganisms and subject them to an array of killing mechanisms, both non-oxidative and oxidative [7]. Because vitamin C is a potent antioxidant [8], it is generally thought that its presence in neutrophils protects them from products of the oxidative burst [9]. However, other potentially relevant immune activities of vitamin C are possible, such as negative regulation of the transcription factor hypoxia-inducible factor 1 (HIF-1) [10]. There is evidence from *in vitro*, animal and human intervention studies supporting a role for vitamin C in the functioning of neutrophils [11,12,13,14,15,16,17,18]. In scorbutic guinea pigs, vitamin C deficiency has been associated with decreased neutrophil oxidative burst and bactericidal activity [13,14,15]. Human studies in individuals with genetic defects in neutrophil function have shown decreases in infectious episodes following vitamin C supplementation, as well as possible improvements in the bactericidal activity of their neutrophils [16,17,18]. The effect of normal dietary intakes of vitamin C on neutrophil functions in healthy individuals has been underexplored.

In addition to phagocytosis, neutrophils have recently been shown to effect extracellular killing through the formation of neutrophil extracellular traps (NETs) [19], a structural DNA framework dotted with microbicidal neutrophil granule proteins and histones [19]. The entire assembly is ejected from the cell and can capture and kill microbes. The production of NETs is a form of cell death which is distinct from apoptosis and necrosis [20]. Expulsion of the NET occurs during plasma membrane rupture, so microbial killing takes place after the neutrophil itself is dead. Vitamin C has recently been shown to be a regulator of NET formation in a model of experimental sepsis [11].

Neutrophils usually die within hours of activation, and this can occur by various mechanisms [21,22]. Neutrophils that have completed phagocytosis and microbial killing undergo phagocytosis-induced cell death, a process similar to apoptosis but which lacks caspase activation [23,24]. They are engulfed by macrophages and removed in a way that prevents the release of damaging cytotoxic cell contents and microbial debris [25]. Unstimulated neutrophils are similarly cleared, undergoing classical apoptosis; their average lifespan is 1–2 days, and roughly 10^10^–10^11^ cells are disposed of daily [26]. We have previously demonstrated in an animal model that vitamin C deficiency prevents the apoptosis and clearance of neutrophils, resulting instead in necrotic cell death [12]. In the current study, we have supplemented a cohort of young adult males with vitamin C-rich *SunGold* kiwifruit and investigated the effect on neutrophil vitamin C status, and four key functions of these cells: chemotaxis, oxidant generation, NET production and apoptosis.

## 2. Study Protocols

### 2.1. Participants

All procedures involving human participants were approved by the New Zealand Health and Disability Ethics Committee (#13/STH/105/AM01). The study was registered with the Australian New Zealand Clinical Trials Registry (ACTRN12613000989741).

Participants were recruited from local tertiary institutions and from our previous vitamin C study databases. Sample size analysis using neutrophil data from our previous vitamin C bioavailability study [27] indicated that, at 90% power and alpha = 0.05, 14 participants would be required (with allowance for 20% withdrawal) in order to detect an effect size of 1.3. A screening interview was carried out with a total of 35 individuals to ascertain eligibility: inclusion criteria (all required for inclusion) included male aged 18–35 years, non-smoker, plasma vitamin C levels <50 μmol/L, and residing in Christchurch for the duration of the study; exclusion criteria (only one required for exclusion) included taking prescription medication (within past three months), allergy/intolerance to kiwifruit, recent smoker (within previous year), taking vitamin C supplements (within past three months), high fruit/juice & vegetable consumption (≥5 servings/day), excessive alcohol consumption (>21 standard drinks/week), diabetes mellitus, bleeding disorders, fainting due to fear of needles. Anthropometric measurements were performed to determine BMI and a fasting venous blood sample was taken to determine plasma vitamin C levels (Table 1). Fourteen volunteers with plasma vitamin C levels <50 μmol/L were enrolled in the study. They provided written informed consent.

**Table 1:** Characteristics of individuals enrolled for the study. Data represent mean ± SD.

	Screened	Enrolled
**Number**	35	14 (12) *^a^*
**Age (years)**	22 ± 4	23 ± 4
**Weight (kg)**	79 ± 15	77 ± 13
**Height (cm)**	180 ± 7	179 ± 6
**BMI (kg/m^2^)**	24 ± 4	24 ± 4
**Plasma Vitamin C (μmol/L)**	55 ± 17	39 ± 12

*^a^* Two of the enrolled participants were excluded from data analysis: one missed the baseline measurements and was subsequently withdrawn, and the other withdrew prior to post-intervention measurements.

### 2.2. Study Design

**Figure 1:**
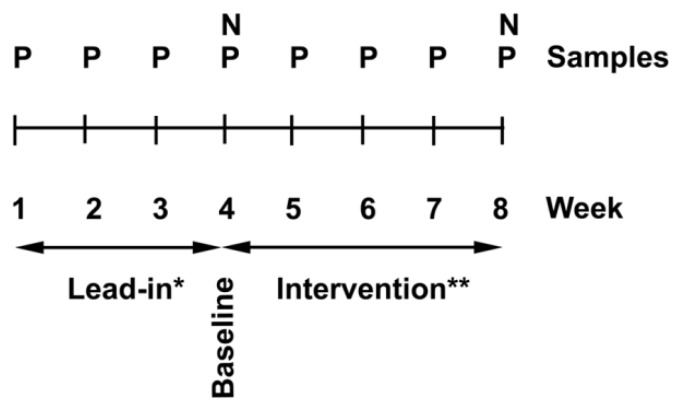
Study design; a three-week lead-in phase to stabilise vitamin C intake (*), was followed by a four-week intervention with two *SunGold* kiwifruit/day (**). Plasma vitamin C levels (P) were monitored weekly and neutrophils (N) were collected at baseline and post-intervention; these were analysed for vitamin C content and neutrophil functional studies were carried out.

Participants with a low baseline vitamin C status were required for this study. Therefore, a lead-in phase was included to allow participants to eliminate juice and high vitamin C foods such as citrus and kiwifruit from their diet, and this regime was maintained for the duration of the study. We have previously shown this to be an effective method for reducing baseline vitamin C levels prior to beginning intervention [27]. Enrolled participants provided weekly fasting blood (4 mL) to monitor plasma vitamin C levels. At week 4, following the lead-in period, extra blood (30 mL) was drawn and neutrophils were isolated for the “baseline” measurements. The participants were provided with Zespri *SunGold* kiwifruit each week for the following four weeks and were asked to consume 2/day. At week 8, the extra blood collection was repeated for the “post-intervention” measures (Figure 1).

### 2.3. Intervention

Gold kiwifruit (*Actinidia chinensis var SunGold*) were provided by Zespri International Ltd. (Tauranga, New Zealand (NZ)) and were stored at ≤4 °C. The vitamin C content of the fruit was measured by high-performance liquid chromatography (HPLC). Analysis indicated that the fruit contained 162 ± 8 mg vitamin C per 100 g fruit (*n* = 5). During the intervention period, participants consumed two kiwifruit/day, without the skin, for four weeks. The actual amount of vitamin C consumed from two peeled kiwifruit was estimated to be ~259 mg (*i.e.*, ~78% of 103 ± 4 g fruit).

### 2.4. Materials

Vacutainer tubes were obtained from Becton Dickinson (Auckland, NZ) and heparin was acquired through the Christchurch Hospital Pharmacy. Cell culture reagents (RPMI 1640 medium, foetal bovine serum (FBS), penicillin and streptomycin), Sytox Green™ and the Apotarget Annexin V-FITC/propidium iodide (PI) kit were supplied by Life Technologies (Auckland, NZ). Ficoll-Hypaque was purchased from Global Sciences (Auckland, NZ) and Calcein AM was from In Vitro Technologies (Auckland, NZ). All other reagents (dextran, glucose, calcium chloride, magnesium chloride, perchloric acid (PCA), diethylenetriaminepentaacetic acid (DTPA) cytochrome *c*, phorbol 12-myristate 13-acetate (PMA), catalase and formyl-methionyl-leucyl-phenylalanine (fMPL) were from Sigma-Aldrich (Auckland, NZ).

### 2.5. Blood and Plasma Collection

Venous blood was collected into 4 mL K_3_-EDTA vacutainer tubes and kept on ice. Samples were centrifuged at 4 °C and the plasma collected and kept on ice for processing prior to vitamin C analysis. For neutrophil isolations, venous blood (30 mL) was collected into tubes containing heparin (15 USP units/mL blood) and kept at room temperature throughout the neutrophil isolation process.

### 2.6. Neutrophil Isolation

Neutrophils were prepared from whole blood using Ficoll-Hypaque, dextran sedimentation and hypotonic erythrocyte lysis as described previously [28]. Isolated neutrophils were counted using a haemocytometer and resuspended in Hank’s buffered saline solution (HBSS; 1 mg/mL glucose, 1 mM calcium chloride, 500 μM magnesium chloride in phosphate buffered saline) and maintained at room temperature.

### 2.7. Vitamin C Analysis

Plasma was mixed with an equal volume of 0.54 M PCA containing the metal chelator DTPA (100 μM), on ice, then pelleted to remove protein. Supernatants were stored at −80 °C prior to analysis by HPLC with electrochemical detection as described previously [29]. Neutrophils (5 × 10^6^ cells, in duplicate) were resuspended in PBS and lysed in an equal volume of 0.54 M PCA/DTPA solution on ice then pelleted to remove protein and the supernatants stored at −80 °C until HPLC analysis.

### 2.8. Chemotaxis Assay

The rate of neutrophil chemotaxis was measured using a method described by Kuijpers *et al* [30]. RPMI 1640 culture medium (225 μL/well), with or without chemoattractant fMLP (100 nM) was added to the wells of a 96-well Fluoroblok™ microtitre plate (Becton Dickinson, Auckland, NZ)). Neutrophils were labelled with Calcein AM (1 μM) in PBS containing glucose (5 mM) for 30 min at 37 °C. They were then washed, resuspended at 1 × 10^6^/mL, and 50 μL was delivered to each insert (0.3 μm pore size). Directed cell migration into the lower chamber was fluorescently monitored at 37 °C (excitation 485 nm, emission 535 nm) over a 40 min period and the maximum rate calculated as a fold increase over the basal level.

### 2.9. Superoxide Production

The rate of superoxide generation by activated neutrophils was measured indirectly as a function of cytochrome *c* reduction [31]. Neutrophils (0.5 × 10^6^ cells) were stimulated with PMA (100 ng/mL) in the presence of catalase (200 μg/mL) and cytochrome *c* (40 μmol/L) and the change in absorbance at 550 nm was recorded over 10 min at 37 °C. The concentration of superoxide was calculated as μmol superoxide min^−1^ per 10^6^ cells (extinction coefficient; 21.1 × 10^3^ M^−1^cm^−1^).

### 2.10. Neutrophil Extracellular Trap Production

NET production was determined using a plate assay method described by Parker *et al* [32]. Neutrophils (0.5 × 10^6^/mL) were resuspended in RPMI 1640 medium, without phenol red, containing 2% FBS and aliquoted into a black 96-well tissue culture plate (six replicates). Cells were incubated in the presence or absence of PMA (20 nM) for 4 h at 37 °C with 5% CO_2_. After 4 h, cells were stained with Sytox Green™ according to the manufacturer’s instructions and the fluorescence measured (excitation 485 nm, emission 520 nm) using a Varioskan™ plate reader (Thermo Scientific, North Shore City, NZ). The amount of NET production was calculated as the difference in fluorescence between stimulated and unstimulated cells at 4 h.

### 2.11. Apoptosis Assay

Neutrophils were resuspended at 1 × 10^6^/mL in RPMI 1640 medium containing 25 U/mL penicillin and 25 μg/mL streptomycin and incubated at 37 °C in a 5% CO_2_ atmosphere. At 0 h and 24 h, samples (1 × 10^6^ cells) were washed and resuspended in buffer containing Annexin V-FITC (as a marker of apoptosis) and PI (as an indicator of necrosis) according to the manufacturer’s instructions. The fluorescence of 10,000 cells was analysed by flow cytometry (Cytomics FC 500 Flow Cytometry system, Beckman Coulter Inc., North Shore City, NZ).

### 2.12. Statistical Analysis

Data are presented as mean ± SEM, unless stated otherwise, and analysis of paired data was done using two-tailed Student’s *t*-test with significance determined as *p* < 0.05 (Sigma Plot v11.0, Systat Software Inc., San Jose, CA, USA).

## 3. Results

### 3.1. Plasma & Neutrophil Vitamin C Levels

Plasma vitamin C levels were monitored weekly and rose to saturation (79 ± 3 μmol/L) after one week of supplementation with two kiwifruit/day. Mean levels remained above 70 μmol/L for the duration of the study. Neutrophil vitamin C levels were measured at baseline (week 4) and after the four-week supplementation interval (week 8). We observed a significant increase in neutrophil vitamin C levels (Table 2).

**Table 2:** Vitamin C content of plasma and neutrophils at baseline and following intervention.

	Baseline	Post-Intervention	*p* Value
**Plasma Vitamin C (μmol/L)**	26 ± 3	72 ± 2	<0.001
**Neutrophil Vitamin C (nmol/10^8^ cells)**	21 ± 1	26 ± 2	0.016

Data represent mean ± SEM, *n* = 12.

### 3.2. Neutrophil Chemotaxis

**Figure 2:**
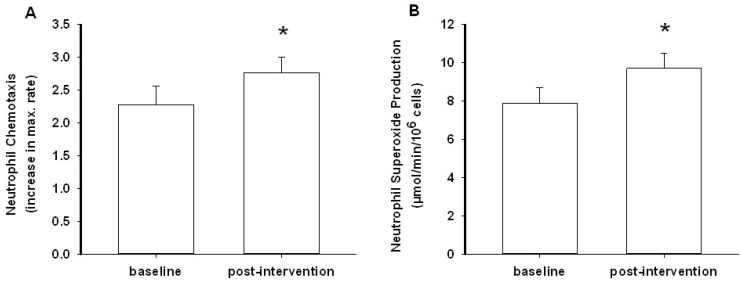
Neutrophil chemotaxis (**A**) and superoxide generation (**B**) at baseline and post-intervention. Data represent mean ± SEM (*n* = 12 and 11), * = *p* < 0.05.

In this study, we used fMLP as a physiologically relevant neutrophil chemoattractant [33]. We monitored the rate of directed neutrophil migration (chemotaxis) and calculated the maximum initial rate over 10 minutes. This revealed a statistically significant 20% increase in the rate of chemotaxis following the kiwifruit intervention (*p* = 0.041, Figure 2A). Supplementation had no effect on control (non-stimulated) neutrophil migration (*p* = 0.500).

### 3.3. Neutrophil Superoxide Production

Measurement of superoxide production is a useful gauge of the potential microbicidal activity of neutrophils [7]. The phorbol ester PMA was used as an optimal stimulant of the superoxide-generating capacity of the cells. Our data showed a 23% increase in superoxide production post-intervention (*p* = 0.031, Figure 2B). The neutrophil sample from one individual was withdrawn from the analysis as flow cytometry analysis revealed an unusually high proportion of lymphocytes. Unlike neutrophils, lymphocytes do not produce superoxide, and this would affect the estimate of neutrophil oxidant production. The results suggest a possible role for vitamin C in enhancing the neutrophil oxidative burst.

### 3.4. NET Production

We measured the extent of NET production in neutrophils from baseline and post-intervention samples using a DNA-specific fluorescent probe, Sytox Green, after incubation in the presence or absence of a stimulant (PMA). We did not observe any effect of kiwifruit supplementation on NET production (Table 3).

### 3.5. Neutrophil Apoptosis

We investigated the effect of the kiwifruit intervention on neutrophil apoptosis by measuring the extent of spontaneous apoptosis at 24 h in unstimulated neutrophils, using flow cytometry with an antibody specific to phosphatidylserine exposed on the cell surface. We did not see any effect of supplementation on neutrophil apoptosis levels (Table 3).

**Table 3:** Neutrophil extracellular trap (NET) production and spontaneous apoptosis (at 24 h) at baseline and following intervention.

	Baseline	Post-Intervention	*p* Value
**NET production (fold increase)**	15 ± 2	17 ± 2	0.49
**Spontaneous apoptosis (% total cells)**	66 ± 4	65 ± 4	0.76

Data represent mean ± SEM, *n* = 12.

## 4. Discussion

Mature peripheral blood neutrophils are primarily responsible for defence against microbial infection and they have the key functions of migration to the site of infection (chemotaxis) and the phagocytosis and killing of microorganisms. Neutrophil clearance is achieved following apoptosis or pathogen-induced cell death, the latter being important for the resolution of inflammation. Neutrophils typically contain high (mM) concentrations of vitamin C, suggesting a critical role for the vitamin in some or all of these functions [34]. In support of this premise, intracellular levels of vitamin C increased by up to 30-fold, mainly through DHA uptake, during bacterial phagocytosis [5]. The traditional belief is that the vitamin C is present at these high concentrations to protect neutrophils from products of the oxidative burst, however, increasing evidence points to other functions [11,12]. The purpose of the current study was therefore to carry out functional studies with human neutrophils to determine the effect of vitamin C supplementation on neutrophil chemotaxis, oxidant production, extracellular trap formation and apoptosis in healthy individuals. We showed that a number of important functional parameters were affected in cells following supplementation of sub-optimal participants with a dietary intake of vitamin C known to provide optimal plasma and leukocyte levels [29].

In this study, we saw a statistically significant increase in both plasma and neutrophil vitamin C levels following intervention with two kiwifruit/day, as we have shown previously [35]. Although the uptake of vitamin C into neutrophils was less than we previously observed, likely due to higher baseline levels, we nevertheless saw effects of the intervention on neutrophil function. There was a statistically significant increase in chemotaxis. The first, and arguably most important, function of neutrophils is to locate, and migrate to, sites of infection. Neutrophils are attracted to sites of inflammation by cytokines that are released from activated immune and endothelial cells, resulting in binding and transmigration of the neutrophils across the endothelium [36]. Once in the tissues, neutrophils follow a chemotactic gradient of molecules emanating from the site of infection. Our findings indicate that kiwifruit supplementation of individuals with low vitamin C status may improve their neutrophil function by increasing their ability to respond to chemotactic stimuli. Two early studies carried out with normal volunteers and with cases of chronic granulomatous disease showed enhanced neutrophil chemotaxis following supplementation with high dose synthetic vitamin C [18,37].

To kill microbes neutrophils trigger an oxidative burst within the phagosome, generating reactive oxidants such as superoxide, hydrogen peroxide, hypochlorous acid and chloramines, that are highly reactive and largely responsible for microbicidal activity [7]. *In vitro* studies have shown enhanced superoxide generation by isolated neutrophils incubated with vitamin C and stimulated with either PMA or fMLP [38,39]. In our intervention study, we observed increased superoxide production following supplementation of the study participants, and this would likely translate into enhanced microbial killing [40]. Others have observed decreased bacterial killing by neutrophils from scorbutic guinea pigs [13,14] and enhanced bacterial killing in individuals with compromised oxidant production following supplementation with vitamin C [17,18]. Although some early studies have shown enhanced hexose monophosphate shunt activity following supplementation with vitamin C [17,18], the mechanism(s) by which vitamin C enhances superoxide production are uncertain and could include effects on expression of the different oxidase subunits, or post-translational modifications of these subunits.

NETs perform in a similar way to phagosomes in that they subject captured pathogens to high, localised concentrations of killing agents. They are abundant at inflammatory sites [41] and are released during the death of neutrophils by NETosis, which is followed by clearance of the neutrophils by macrophages [42]. It is of interest to note that NETosis is dependent on oxidant generation [20]. A recent *Gulo* knockout mouse study showed that vitamin C was a negative regulator of NET formation in experimental sepsis, and that it protected against tissue injury caused by excessive NETosis [11]. Formation of NETs is an active process and a recent study has suggested it may be dependent on the transcription factor HIF-1 [43]. Since vitamin C is a cofactor for the hydroxylases that regulate HIF-1α protein levels and DNA binding [44], this suggests a potential mechanism by which vitamin C could regulate NET generation. We have previously shown that vitamin C-deficient neutrophils from the *Gulo* knockout mouse have elevated HIF-1 activation, but this was only determined with complete deficiency [12]. In the current study, with only a modest increase in neutrophil vitamin C content, we did not observe any effect of supplementation on NET production.

Apoptosis is characterised by nuclear condensation and DNA fragmentation, extensive membrane blebbing, caspase activation and the exposure of phosphatidylserine on the outer cell surface [24,45]. Macrophages have phosphatidylserine receptors with which they identify apoptotic cells for clearance. Our previous work with the *Gulo* knockout mouse model showed that vitamin C was essential for neutrophil apoptosis; complete deficiency inhibited apoptosis and also prevented the uptake of neutrophils by macrophages *in vitro*, resulting in necrotic cell death which, in an *in vivo* setting, could have devastating effects on the tissues [12]. The decreased apoptosis observed in vitamin C deficient neutrophils was associated with up-regulation of HIF-1. HIF-1 drives the transcription of many genes involved in the control of metabolism, cellular stress response and cell survival [46]. Recent studies have shown the potential involvement of HIF-1 in neutrophil and other immune cell function [47,48] and work in HIF-1 phagocytic cell knockout animals has shown possible involvement of HIF-1 in bacterial killing by neutrophils [47]. In our current study, we did not observe any effect of supplementation on spontaneous neutrophil apoptosis.

There are several possible explanations for our findings of an effect of vitamin C supplementation on chemotaxis and oxidant production, but not on NET generation or apoptosis. Firstly, the vitamin C requirements of different neutrophil functions may vary. Chemotaxis occurs at an early stage, before the oxidative burst, and would thus rely on existing intracellular stores of vitamin C. Therefore, increasing the basal intracellular concentration of vitamin C, as we did with the kiwifruit supplementation, may increase the chemotactic ability of the cell. Similarly, superoxide is produced during the oxidative burst, a process which coincides with the uptake of large quantities of DHA into the neutrophil [5]. Any requirement for vitamin C in the initiation of this process must therefore rely on existing intracellular vitamin C stores and the increase in superoxide generation that we observed *ex vivo* following intervention indicates that this is likely. It is notable that the positive effects we observed were evident in processes triggered prior to the generation of large quantities of oxidants. It is therefore possible that downstream functions, such as NET production and apoptosis, may have an increased requirement for vitamin C, which is usually met by the import of DHA during phagocytosis and its subsequent intracellular reduction to vitamin C. However, in our experimental system, this could not occur because the neutrophils were incubated in cell culture medium which lacks vitamin C. As a result, the cells would be unable to take up additional vitamin C and any beneficial *in vivo* effects of the intervention would not be evident. The amount of vitamin C required to support optimal apoptosis is not yet known as our previous study used completely vitamin C deficient neutrophils [12].

We and others have previously shown that unstimulated neutrophils become saturated at relatively low dietary intakes of vitamin C (~100 mg/day) [2,35]. Although we did see a statistically significant increase in neutrophil vitamin C levels following intervention, it is possible that our study cohort were not sufficiently depleted at baseline to impair neutrophil apoptotic function enough to observe an effect following supplementation. Our earlier *Gulo* knockout mouse study, in which we were able to obtain vitamin C depleted neutrophils, indicates that vitamin C is critical for neutrophil apoptosis and clearance from sites of inflammation *in vivo* [12]. The effect of vitamin C supplementation on the apoptosis of activated neutrophils, which is dependent on products of the oxidative burst, has not yet been assessed.

Kiwifruit are an outstanding source of vitamin C, however, they also contain reasonable levels of other important micronutrients, such as Vitamins E, B and K, carotenoids and the minerals copper and magnesium [49]. These micronutrients could potentially influence neutrophil function, either individually or in concert with vitamin C [50]. In a previous kiwifruit intervention study we assessed the intake and circulating and tissue levels, of vitamins C and E and carotenoids, and found that, other than a small increase in vitamin E intake, vitamin C was the only micronutrient that increased dramatically following supplementation with kiwifruit; no increase of carotenoid intake or tissue levels was observed [51]. Kiwifruit also contain numerous different bioflavonoids, particularly in their skins [52], which could potentially influence neutrophil function [53]. However, the skins were not consumed by our study participants, and the *in vivo* bioavailability of many bioflavonoids is limited [54], thus diminishing potential effects on neutrophil function. Therefore, it is likely that the observed effects of kiwifruit consumption on neutrophil function in our study are predominantly due to the high vitamin C content of the fruit.

## 5. Conclusions

Overall, our study showed that supplementation with vitamin C-rich *SunGold* Kiwifruit is associated with a significant increase in neutrophil vitamin C status and the important anti-microbial functions of chemotaxis and oxidant production. We saw no effect of supplementation on NET generation or apoptosis; these functions may rely on the continuous presence of vitamin C, which would be observed *in vivo*, but not under cell culture assay conditions. Alternatively, it is possible that in the current study, the neutrophils had adequate vitamin C levels at baseline for these functions. This suggests that more in-depth investigations into the effect of vitamin C on neutrophil function are warranted, particularly in individuals with depleted vitamin C status, such as critically ill patients [55,56].
